# Feature fusion based transformer for sentiment analysis in social networks

**DOI:** 10.1371/journal.pone.0333416

**Published:** 2025-11-07

**Authors:** Shiyong Li, He Li, Juan Du, Shitao Yan, Chuang Dong

**Affiliations:** 1 School of Computer Science and Technology, Henan Institute of Science and Technology, Xinxiang, China; 2 Henan Intelligent Emergency Service and Security Engineering Research Center, Nanyang Normal University, Nanyang, China; 3 Business School, Xinxiang University, Xinxiang, China; Philadelphia University, JORDAN

## Abstract

Sentiment analysis methods aim to evaluate users’ mental health conditions by analyzing their posted content (text, images, and audio) on social networks. However, given the diversity and complexity of social media information, traditional single-modal sentiment analysis techniques exhibit limitations in accurately interpreting users’ emotional states and may even lead to contradictory conclusions. To address this challenge, this paper proposes a Feature Fusion Based Transformer (FFBT) solution. The framework consists of three key steps: Firstly, RoBERTa and ResNet50 models are employed to extract features from textual and image data in social media posts, respectively. Then, a multimodal Transformer architecture facilitates feature alignment and fusion across different modalities. Finally, the fused features are fed into a fully connected network (FCN) for sentiment classification, ultimately determining the user’s emotional state. Experiments conducted on a custom dataset constructed from social media platform data demonstrate that FFBT outperforms existing sentiment analysis algorithms by 4.1% in accuracy and 5% in F1-scores, respectively.

## 1 Introduction

**Fig 1:**
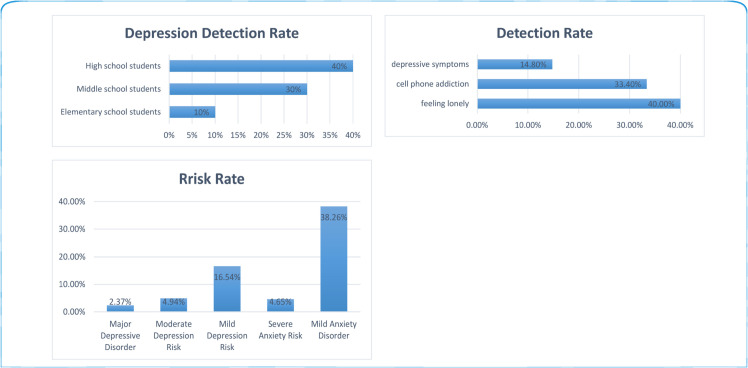
The 2023 Blue Book of China’s Mental Health unveils the alarming state of students’ mental health, which urgently requires intervention from the education system.

In the digital era, the accelerated pace of modern life has emerged as a pivotal factor driving the dramatic escalation of psychological stress, consequently elevating the incidence of mental health disorders such as depression. According to authoritative data from *The 2023 Blue Book of China’s Mental Health* (Fig 1), adolescents’ mental health status in China exhibits significant variations across educational stages: The basic education phase demonstrates a progressive increase in depression detection rates (10% in primary, 30% in junior high, and 40% in senior high schools), while university students display 16.54% prevalence of mild depression and 4.94% for severe cases. Notably, anxiety disorders present even more prominent risks, with 38.26% mild, 4.65% moderate, and 2.37% severe manifestations. Meanwhile, driven by traffic incentives, social media platforms systematically amplify anxiety-provoking content through algorithmic mechanisms. This “emotional amplification effect” has transformed these platforms into primary channels for emotional catharsis, where users increasingly express their feelings through various modalities, including textual posts, visual imagery, and short videos. Under this context, utilizing emotional expression data from social platforms to assess students’ mental health status or affective states, thereby enabling personalized psychological counseling and education, has emerged as a critical research topic. In recent years, researchers have employed diverse intelligent algorithms for personalized psychological education [1], online depression screening [2], and opinion mining on social platforms [3], assisting professionals in delivering more effective mental-health prevention and counseling.

Yu et al. [4] designed a label generation module based on a self-supervised learning strategy to automatically obtain independent unimodal supervision signals. Subsequently, by jointly training multimodal and unimodal tasks, they learned effective representations for multimodal scenarios. Yao et al. [5] argued that human language is inherently multimodal, integrating natural language, facial movements, and acoustic behaviors. They proposed a Multimodal Transformer (MulT) for sentiment analysis, which captures interactions between multimodal sequences at different time steps and implicitly aligns one modality’s sequence to another. Hazarika et al. [6] introduced the MISA framework for sentiment analysis, mapping each modality into two subspaces: a modality-invariant subspace and a modality-specific subspace. These representations together provide a comprehensive view of multimodal data and are fused for sentiment prediction. Zadeh et al. [7] modeled multimodal sentiment analysis as a joint task of intra-modal dynamics and inter-modal dynamics, proposing a novel Tensor Fusion Network (TFN) for this purpose.

These algorithms have achieved notable results and advanced sentiment analysis, but they also exhibit certain limitations. On one hand, traditional unimodal feature extraction techniques (e.g., Fine-tuning CNNs [8], LCA-BERT [9]) struggle to comprehensively capture deep semantic features in text or effectively extract fine-grained visual features in images. On the other hand, traditional multimodal fusion techniques (e.g., TFN [7], MulT [5]) directly fuse full-modal features, leading to suboptimal performance on cross-modal datasets. Multimodal sentiment analysis faces two major challenges:

1. **Unimodal encoders fail to extract sufficiently fine-grained features**, limiting their discriminative power.
2. **Inadequate cross-modal feature alignment**, which can lead to polarity reversal (e.g., conflicting sentiment signals between text and images shown in Table 1).

**Table 1:** The combination of text and different emojis may demonstrate opposed emotions.

No.	Text & Image Data	Sensitive
1	Oh, yes, I am happy. (with emoji of face-with-rolling-eyes)	negative
2	Oh, yes, I am happy. (with emoji of grimacing-face)	positive

To address the aforementioned challenges in multimodal sentiment analysis, this paper proposes a **F**eature **F**usion- **B**ased **T**ransformer (FFBT) for multimodal sentiment analysis. The approach first encodes textual inputs with RoBERTa [10] and extracts image features via ResNet50 [11]. Next, cross-modal multi-head attention aligns the two modalities, after which the aligned features are fed into a multimodal Transformer encoder to intensify inter-modal interactions and achieve deep feature fusion. Finally, the fused representations are passed through fully connected layers to accurately predict the sentiment category of the multimodal content. The key contributions of the proposed FFBT method are summarized below:

1. **High-quality unimodal encoders**: RoBERTa is employed as the text encoder, leveraging its bidirectional Transformer and dynamic masking to fully capture contextual semantics; ResNet50 serves as the image encoder, extracting 2048-dimensional fine-grained image features via residual blocks and global average pooling.
2. **Cross-modal feature alignment**: After unimodal extraction, a cross-modal Transformer encoder [5] explicitly aligns text and image features, enabling the model to capture intricate interactions such as “sarcastic emoji vs. positive text.” A cosine-embedding contrastive loss is further employed to pull together matched image–text pairs and push apart mismatched ones in the shared space, refining alignment and sharpening the model’s discrimination of fine-grained emotional nuances.

Leveraging data from social-network platforms, this paper constructs a multimodal dataset that contains both text and images, named **S**ocial **D**ata in **T**ext and **I**mage (SDTI). Experimental results demonstrate that the proposed method achieves higher accuracy (+4.1%) and F1-score (+5%) compared to baseline algorithms.

The remainder of this paper is organized as follows: Sect 2 reviews related work on multimodal sentiment analysis; Sect 3 outlines the overall pipeline of the FFBT approach; Sect 4 details the design specifics and enhancements of FFBT; Sect 5 presents the experiments conducted on SDTI together with their results; and Sect 6 concludes the paper and discusses future directions.

## 2 Related works

This section briefly surveys the work of the predecessors in sentiment analysis, covering both single-modal and multimodal approaches.

### 2.1 Single-modal sentiment analysis

Chen et al. [12] proposed a simple framework for contrastive learning of visual representations, advancing computer vision by enabling models to learn robust features without labeled data through positive and negative sample training.

Tran et al. [13] examined spatiotemporal convolutions in action recognition, showing they effectively capture video motion information and improve recognition accuracy.

Carreira and Zisserman [14] introduced a two-stream model for action recognition and the Kinetics dataset, achieving top performance by integrating spatial and temporal information.

Kugate et al. [15] developed an efficient method for video key frame extraction using CNNs and clustering, identifying representative frames for applications like video summarization.

Fan et al. [16] presented a hybrid CNN-RNN model for video emotion recognition, capturing spatial and temporal features to achieve state-of-the-art performance.

Pang et al. [17] used dynamic graph convolutional networks for aspect-based sentiment analysis, updating graph structures to capture aspect relationships better and improve classification accuracy.

Li et al. [18] employed dual graph convolutional networks to model aspect interconnections in sentiment analysis, achieving top results through enhanced sentiment categorization precision.

Although the aforementioned studies have advanced emotion recognition, single-modal approaches remain confined within their respective modalities and thus cannot fully convey the richness of human affect. In reality, users on social media rarely rely on a single data type when posting content. Consequently, this paper adopts a multimodal framework for emotion recognition.

### 2.2 Basic multimodal fusion for sentiment analysis

As shown in Table 1, different images paired with the same text data may exhibit entirely different emotional tendencies. Sometimes, a purely narrative text cannot be accurately judged for sentiment, but when paired with a “joyful” emoji, the sentiment tendency of the text + image data can be easily determined. Therefore, single-modal data is not sufficient to make an accurate sentiment judgment, and sentiment analysis must be conducted on multimodal data to achieve an ideal state. Driven by the demand for more accurate emotion understanding, researchers have developed many general-purpose multimodal datasets and baseline models.

Busso et al. [19] introduced the IEMOCAP dataset, which includes multimodal data like audio, video, and text from emotionally charged actor interactions, providing detailed emotion annotations for model training and evaluation.

Poria et al. [20] presented the MELD dataset, designed for conversational emotion recognition, containing multimodal data from multi-party conversations with detailed emotional state annotations, effectively training advanced emotion recognition models.

Abdullah et al. [21] explored deep learning for multimodal emotion recognition, developing a model integrating text, audio, and video features for diverse contexts, matching existing approaches’ performance.

Building upon these baseline models, researchers have further explored a variety of general-purpose multimodal fusion strategies.

Gupta et al. [22] proposed Visatronic, a multimodal speech synthesis model combining text and audio features with a decoder-only architecture, significantly improving speech synthesis quality across datasets.

Mai et al. [23] used hybrid contrastive learning for multimodal sentiment analysis, combining text, audio, and video features to enhance sentiment classification accuracy through modality alignment.

Zadeh et al. [7] developed the Tensor Fusion Network (TFN), using a tensor fusion layer to merge text, audio, and video features, outperforming existing methods in multimodal and unimodal sentiment analysis.

Liu et al. [24] introduced an efficient multimodal fusion approach using modality-specific factors, reducing parameters while maintaining high accuracy in sentiment analysis.

Kakuba et al. [25] extend conventional speech-based emotion recognition by proposing a deep-learning multi-learning framework termed DBMER. Integrating CNNs, RNNs, and multi-head attention, DBMER significantly outperforms traditional multimodal methods in both accuracy and robustness.

### 2.3 Advanced multimodal fusion for sentiment analysis

In recent years, to more precisely determine the emotional categories expressed by diverse modalities, researchers have shifted toward context- and causality-enhanced fusion strategies and have achieved notable progress.

Poria et al. [26] focused on context-dependent sentiment analysis in user-generated videos, proposing a model considering video content context to improve sentiment classification accuracy.

Xing et al. [27] proposed an adapted dynamic memory network for conversational emotion recognition, dynamically updating memory based on conversation context to better capture emotional states.

Hazarika et al. [28] introduced ICON, an interactive conversational memory network for multimodal emotion detection, using a memory network to grasp conversation context and enhance emotion recognition precision.

Xiao et al. [29] proposed Atlantis, an aesthetic-oriented multi-granularity fusion network for joint multimodal aspect-based sentiment analysis, combining text, image, and aesthetic features for state-of-the-art performance.

Yue et al. [30] developed Knowlenet, a knowledge fusion network for multimodal sarcasm detection, combining text, image, and knowledge graph features to significantly improve sarcasm detection accuracy.

Other researchers have conducted more detailed studies from perspectives such as graphs, hypergraphs, counterfactual interventions, and polarity, proposing more fine-grained multimodal fusion methods.

Since cross-modal attention tends to overlook modality-specific cues when distinguishing similar samples, Huang et al. [31] devise a cross-sample fusion strategy: features from different samples are combined, and adversarial training coupled with pairwise-prediction tasks is employed to preserve fine-grained modality-specific information.

Traditional multimodal models often neglect causal relations between modalities, giving rise to spurious correlations and underperforming cross-modal attention. To overcome these limitations, Huang et al. [32] propose the Attention-based Causality-Aware Fusion (AtCAF) network. First, the Causality-Aware Text Debiasing Module (CATDM) is introduced with a front-door adjustment to capture debiased textual causal representations. Second, a Counterfactual Cross-modal Attention (CCoAt) module is employed to inject causal information during fusion, yielding aggregated representations that are causally consistent.

Chen et al. [33] tackle the optimization imbalance inherent in multimodal learning by introducing the Adaptive Gradient-Scaling & Sparse Mixture-of-Experts model (AGS-SMoE). The framework devises a gradient-scaling policy that equalizes the training dynamics across heterogeneous encoders. In addition, it integrates a Sparse Mixture-of-Experts (SMoE) mechanism that sparsely perceives and processes multimodal tokens according to their current contention states, thereby mitigating redundant computation and enhancing efficiency.

Li et al. [34] argue that conventional multimodal retrieval systems fall short of educational demands for instructional slide retrieval. They propose EduCross, a framework that introduces Dual-Channel Adversarial Bipartite Hypergraph Learning, which combines a generative adversarial network with text–image dual channels to achieve precise bidirectional graph–text mapping.

Ramkrishnan et al. [35] extracted seven key acoustic features from audio datasets and evaluated each feature with both traditional classifiers and deep-learning models (LSTM and CNN). They found that the importance of individual features varies across models, underscoring the necessity of feature optimization in speech-based emotion recognition.

Huang et al. [36] propose a novel approach named Heterogeneous Hypergraph Attention Network with Counterfactual Learning (${\text{H}}^{2}\text{CAN}$). They construct a heterogeneous hypergraph based on emotional-expression features and mitigate bias through a Counterfactual Intervention Task. Additionally, single-modal labels are leveraged to let the model adaptively identify which modality carries bias, further enhancing its ability to handle biased information.

To counteract the text-bias that distorts affective representations in multimodal conversational emotion recognition, Li et al. [37] introduce FrameERC, a new framework that captures crucial emotional cues overlooked by conventional spatial message-passing GNNs. FrameERC amplifies the effective contribution of non-textual modalities in Emotion Recognition in Conversations (ERC), ensuring a more holistic semantic understanding.

Shi et al. [38] propose a novel dialogue-based multimodal emotion-recognition framework that learns a unified semantic embedding from multimodal data, mitigating text-overreliance bias without a marked increase in parameters. A modality-aware extraction module further captures both generic and sensitive information in parallel within the shared multimodal semantic space.

Huang et al. [39] observe that, given a fixed emotional polarity, the perceived emotional intensity increases monotonically with the expressive amplitude. Building on this insight, they propose a Polarity-Aware Mixture-of-Experts network for Multimodal Sentiment Analysis (PAMoE-MSA) that jointly learns polarity-specific and polarity-common representations to explicitly capture the monotonic relationship underlying multimodal emotional expressions.

Compared with the aforementioned previous works, the FFBT method primarily contributes in the following aspects:

1. **Enhanced Context and Cross-modal Interaction**: By employing a multimodal Transformer encoder, it better captures the correlations and contextual relationships between text and images, thereby strengthening the interaction across different modalities.
2. **Improved Robustness to Noisy Data**: Leveraging the inherent robustness of RoBERTa and ResNet50, it effectively handles diverse data types and noise.
3. **High Scenario Adaptability**: Featuring a flexible design paradigm, the framework serves as a versatile foundation for model architecture, demonstrating broad applicability across multiple scenarios.

## 3 Overview

**Fig 2:**
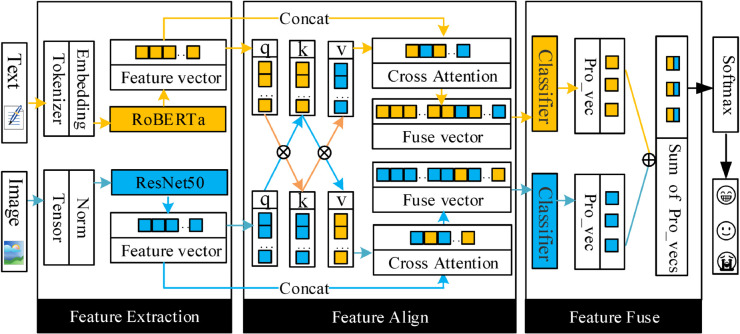
The architecture of the proposed Feature Fusion Based Transformer (FFBT) approach is shown in the figure. After RoBERTa and ResNet50 extract features, and the features are aligned by cross-modal attention, classification can be done as positive, neutral, or negative by FCN with Softmax function.

As illustrated in Fig 2, the proposed architecture comprises three sequential stages.

During the feature extraction stage, the text is first converted into word embedding vectors using subword tokenizers, and then these word embeddings are fed into the RoBERTa model to extract the text’s feature vectors. The image is preprocessed to generate a tensor, which is then passed through the ResNet50 model to obtain the image’s feature vectors.

In the feature alignment stage, the text and image feature vectors first generate their respective projected vectors. Then, through a cross-modal attention mechanism, the text and image features are aligned, producing cross-attention vectors for both text and image. The projected vectors and cross-attention vectors are then concatenated to form the aligned feature vectors for both text and image.

During the feature fusion stage, the aligned feature vectors of the text and image are each passed through a fully connected neural network classifier to obtain their respective probability vectors. The probability vectors of the text and image are combined and then processed through a Softmax function to compute the final probability vector. Finally, based on the maximum value in the probability vector, the model determines the sentiment category to which the given text and image belong.

## 4 Design details

### 4.1 Feature extraction

**Fig 3:**
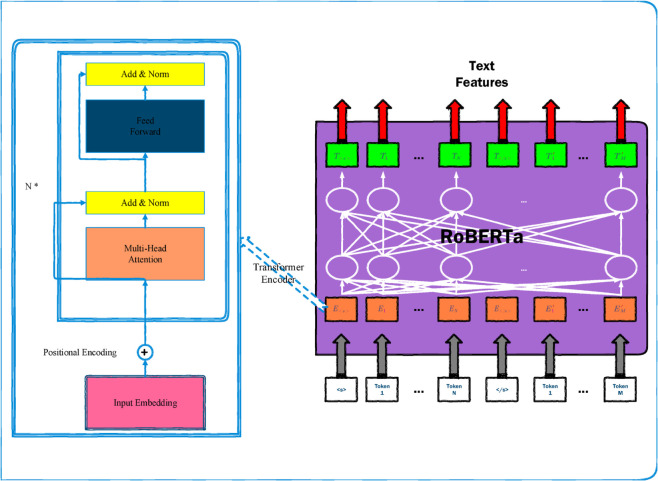
The overall logic architecture of RoBERTa, which has been adapted from the structure of BERT.

1) Compared with image data, text data plays a more crucial role in sentiment classification tasks, as it carries the majority of emotional tendencies when people express feelings. This paper employs the RoBERTa model for text feature extraction, and its overall architectural diagram is illustrated in Fig 3.

When text data is fed into RoBERTa, the model encodes it into a feature vector ${𝐡}_{\text{text}}$.

For a given input text, meaningless symbols are first removed. The cleaned text is then tokenized into a token sequence using a subword tokenizer such as Byte-Pair Encoding. Special tokens are inserted: “CLS” (or “<*s*>”) is prepended to the sequence, and “SEP” (or “</*s*>”) is appended at the end of each sentence.

$${𝐗}_{\text{augmented}}=[<s>,x_{1},\cdots ,</s>,\cdots ,x_{L}]$$ (1)

Where, ${𝐗}_{\text{augmented}}$ denotes the token sequence after adding special tokens, each $x_{i}\in \{1,2,\ldots ,V\}$ and *V* is the vocabulary size; *L* is the length of token sequence.

After the token sequence is fed into the embedding layer, every token is converted to Token Embedding. Position Embedding is then added—without the Segment Embedding used in BERT—to yield the final text input embedding ${𝐄}_{\text{text}}$.

$${𝐄}_{\text{token}}={𝐖}_{\text{token}}\cdot {𝐗}_{\text{augmented}}$$ (2)

$${𝐄}_{\text{pos}}={𝐖}_{\text{pos}}\cdot 𝐏$$ (3)

$${𝐄}_{\text{text}}={𝐄}_{\text{token}}+{𝐄}_{\text{pos}}\in {\mathbb{R}}^{L\times d}$$ (4)

Where ${𝐖}_{\text{token}}\in {\mathbb{R}}^{V\times d}$ is the token-embedding matrix with vocabulary size *V* and embedding dimension *d* (e.g., 768); ${𝐖}_{\text{pos}}\in {\mathbb{R}}^{L\times d}$ is the position-embedding matrix; 𝐏=[0,1,2,…,L−1] is the position-index sequence; ${𝐄}_{\text{token}}$ and ${𝐄}_{\text{pos}}$ denote the token and positional encodings, respectively.

Finally, ${𝐄}_{\text{text}}$ is fed into the Transformer encoder of RoBERTa. The encoder consists of *N* identical layers (usually *N* = 12) stacked on top of each other. Each layer comprises two sub-layers: the first is a multi-head self-attention layer, and the second is a feed-forward neural network. Residual connections are applied around every sub-layer. The core attention mechanism of the Transformer involves three key input matrices— **Q**, **K**, and **V**, which are obtained by multiplying the input ${𝐄}_{\text{text}}$ with three distinct linear projection matrices ${𝐖}_{Q}$, ${𝐖}_{K}$, and ${𝐖}_{V}$, respectively.

$$𝐐={𝐖}_{Q}\times {𝐄}_{\text{text}}$$ (5)

$$𝐊={𝐖}_{K}\times {𝐄}_{\text{text}}$$ (6)

$$𝐕={𝐖}_{V}\times {𝐄}_{\text{text}}$$ (7)

By measuring the relevance between **Q** and **K** with dot products, we obtain an attention-score matrix. A scaling factor *d*_*t*_ is then applied to stabilize the variance of these scores, after which Softmax normalizes them into final attention weights. These weights are multiplied by **V** to produce the updated semantic representation.

$$\mathrm{Attention}(𝐐,𝐊,𝐕)=\mathrm{Softmax}(\frac{𝐐(𝐊{)}^{T}}{\sqrt{d_{t}/m}})𝐕$$ (8)

where *m* denotes the number of attention heads in the multi-head attention.

The semantic representations produced by all *m* attention heads are concatenated, multiplied by an output projection matrix, and then passed through a residual connection followed by layer normalization to yield the self-attention output ${𝐄}_{\text{attn}}^{l}$ at layer l∈[1,N].

$${𝐄}_{\text{attn}}^{l}=\text{LayerNorm}(\text{Concat}({\text{Attention}}_{1}^{l},\ldots ,{\text{Attention}}_{m}^{l}){𝐖}_{O}^{l}+{𝐄}^{l-1})$$ (9)

where ${𝐖}_{O}^{l}$ is the output projection matrix, and ${𝐄}^{l-1}$ denotes the input to the current layer or the output of the preceding layer; specifically, ${𝐄}^{0}={𝐄}_{\text{text}}$.

Next, ${𝐄}_{\text{attn}}^{l}$ is fed into the feed-forward network; after a linear transformation, it yields the feed-forward network’s output ${𝐄}_{\text{ffn}}^{l}$. A residual connection followed by layer normalization is then applied, producing the final output of the *l*-th Transformer encoder layer ${𝐄}^{l}$.

$${𝐄}_{\text{ffn}}^{l}=\text{GELU}({𝐄}_{\text{attn}}^{l}{𝐖}_{1}^{l}+{𝐛}_{1}^{l}){𝐖}_{2}^{l}+{𝐛}_{2}^{l}$$ (10)

$${𝐄}^{l}=\text{LayerNorm}({𝐄}_{\text{ffn}}^{l}+{𝐄}_{\text{attn}}^{l}),l\in [1,N]$$ (11)

Where, ${𝐖}_{O}^{l}$ is the output-projection matrix; ${𝐖}_{1}^{l}$ and ${𝐖}_{2}^{l}$ are the weight matrices of the feed-forward network linear transformation; ${𝐛}_{1}^{l}$ and ${𝐛}_{2}^{l}$ are the bias matrices of the feed-forward network linear transformation; GELU denotes the activation function; LayerNorm is the layer-normalization function.

After processing through all *N* Transformer encoder layers, the final output ${𝐄}^{N}$ is obtained. Averaging the vectors across all positions yields the ultimate textual feature vector ${𝐡}_{\text{text}}$.

$${𝐡}_{\text{text}}=\frac{1}{L}\sum_{i=0}^{L-1}{𝐄}_{i}^{N}$$ (12)

$${𝐡}_{\text{text}}=\{T_{\text{CLS}},T_{1},\cdots ,T_{\text{SEP}},\cdots ,T_{L}\}\in {\mathbb{R}}^{L\times d}$$ (13)

**Fig 4:**
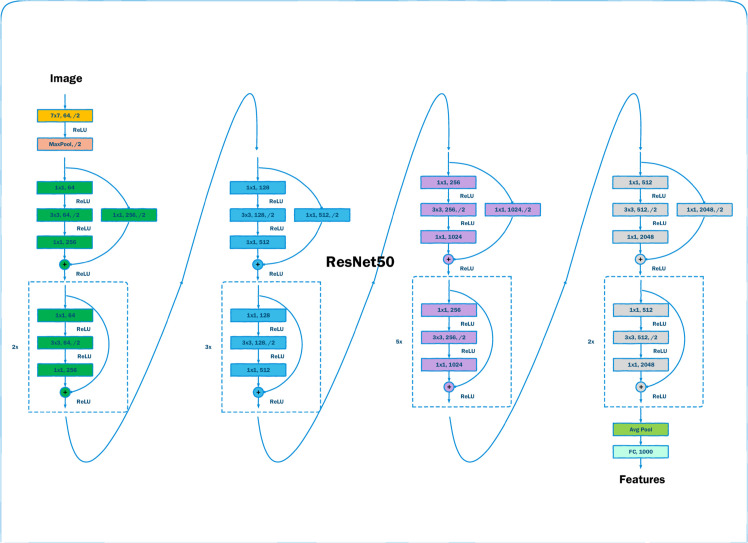
The overall architecture of ResNet50.

2) In sentiment classification tasks, image data provides complementary information to textual data, enabling more precise sentiment analysis. The cross-modal interaction between image and textual data establishes a foundation for subsequent feature fusion models to capture inter-modal semantic relationships. For image feature extraction, we employ the ResNet50 model, whose overall architecture is depicted in Fig 4.

When an image is fed into ResNet50, the model processes it and outputs the feature vector ${𝐡}_{\text{image}}$.

For each image, we first perform preprocessing: resize it to 224 × 224, center-crop, and normalize it, yielding the image input tensor **X**.

$$𝐗\in {\mathbb{R}}^{1\times 3\times 224\times 224}\;(\text{batch\_size}=1)$$ (14)

The image then undergoes an initial convolution and pooling, producing the ResNet50 input tensor **Z**.

$$𝐙=\text{MaxPool}(\text{ReLU}\left(\text{BatchNorm}\left(𝐗\cdot {𝐖}_{\text{conv}}+{𝐛}_{\text{conv}}\right)\right))$$ (15)

where ${𝐖}_{\text{conv}}$ is the convolution kernel matrix with stride 2 and padding 3, and ${𝐛}_{\text{conv}}$ is the corresponding bias vector.

Next, **Z** is processed through ResNet50’s four residual-block groups, yielding the final feature ${𝐙}_{\text{final}}$.

$${𝐙}_{\text{final}}={\mathrm{Layer}}_{M}\left(\cdots {\mathrm{Layer}}_{1}\left(𝐙\right)\cdots \right)\in {\mathbb{R}}^{1\times 2048\times 7\times 7}$$ (16)

Where ${\mathrm{Layer}}_{i},\;i\in [1,M]$ denotes the *i*-th residual-block group in ResNet50. Each group consists of three consecutive convolution layers with kernel sizes 1×1, 3×3, and 1×1. For any such group, the input ${𝐙}_{\text{in}}$ and output ${𝐙}_{\text{out}}$ satisfy the Eqs 17–20 as follows:

$$𝐀=\text{ReLU}\left(\text{BatchNorm}\left({𝐖}_{1\times 1}\cdot {𝐙}_{\text{in}}\right)\right)$$ (17)

$$𝐁=\text{ReLU}\left(\text{BatchNorm}\left({𝐖}_{3\times 3}\cdot 𝐀\right)\right)$$ (18)

$$𝐂=\text{BatchNorm}\left({𝐖}_{1\times 1}\cdot 𝐁\right)$$ (19)

$${𝐙}_{\text{out}}=\mathrm{Layer}({𝐙}_{\text{in}})=𝐂+{𝐙}_{\text{in}}$$ (20)

where ${𝐖}_{1\times 1}$ and ${𝐖}_{3\times 3}$ denote the weight matrices of the 1×1 and 3×3 convolution layers, respectively. **A**, **B**, and **C** represent transitional variables.

Finally, global average pooling is applied to ${𝐙}_{\text{final}}$ to obtain the image feature vector ${𝐡}_{\text{image}}$.

$${𝐡}_{\text{image}}=\mathrm{GlobalAvgPool}({𝐙}_{\text{final}})$$ (21)

where GlobalAvgPool denotes global average pooling.

### 4.2 Feature alignment and fusion

To narrow the gap between modalities and enhance their interaction, we introduce a cross-modal feature-alignment layer, in which the cross-modal component [40] employs a multimodal Transformer encoder to mediate inter-modal communication.

The extracted textual feature vector and the extracted image feature vector are fed into a multimodal Transformer encoder, yielding the cross-modal-enhanced representations ${𝐄}_{\text{cross}}^{t}$ and ${𝐄}_{\text{cross}}^{i}$. The encoder is composed of *N*_*L*_ identical layers stacked one after another, where each layer contains two sublayers: a multi-head cross-modal attention layer and a position-wise feed-forward network. Residual connections surround every sub-layer. Unlike the self-attention described earlier, in cross-modal attention the query matrix **Q** is derived from the current modality *M*_1_, whereas the key matrix **K** and value matrix **V** are derived from the other modality *M*_2_. Except for this cross-modal attention module, the remaining computations of the multimodal Transformer encoder are the same as Eqs 9–11. The cross-modal attention layer is formulated as:

$${𝐐}_{M_{1}}={𝐖}_{Q}^{M_{1}}\times {𝐡}_{M_{1}}$$ (22)

$${𝐊}_{M_{2}}={𝐖}_{K}^{M_{2}}\times {𝐡}_{M_{2}}$$ (23)

$${𝐕}_{M_{2}}={𝐖}_{V}^{M_{2}}\times {𝐡}_{M_{2}}$$ (24)

$$\mathrm{CrossAttention}({𝐐}_{M_{1}},{𝐊}_{M_{2}},{𝐕}_{M_{2}})=\mathrm{Softmax}(\frac{{𝐐}_{M_{1}}({𝐊}_{M_{2}}{)}^{T}}{\sqrt{d_{t}/m}}){𝐕}_{M_{2}}$$ (25)

The cross-modal enhanced representation ${𝐄}_{\text{cross}}$ obtained via the cross-modal attention mechanism cannot be used directly for classification; it must first be converted into a fixed-size feature embedding ${𝐇}_{\text{cross}}\in {\mathbb{R}}^{1\times d_{t}}$ through a pooling operation [41], as shown in the following equation:

$${𝐇}_{\text{cross}}=\mathrm{mean}({𝐄}_{\text{cross}})$$ (26)

The projected representation ${𝐇}_{\text{Projection}}$ and the cross-modal enhanced embedding ${𝐇}_{\text{cross}}$ are concatenated to yield the final fused features for text and image, respectively, as shown in the following equation:

$$𝐇=\mathrm{Concat}({𝐇}_{\text{cross}},{𝐇}_{\text{Projection}})$$ (27)

Finally, each fused feature **H** is passed through a fully-connected layer to produce separate probability vectors ${𝐇}_{\text{text}}^{\prime }$ and ${𝐇}_{\text{image}}^{\prime }$; these two vectors are then summed to obtain the final probability vector ${𝐇}_{\text{final}}$, as shown in the following equation:

$${𝐇}^{\prime }={𝐖}_{\text{fc}}𝐇+{𝐛}_{\text{fc}}\in {\mathbb{R}}^{C}$$ (28)

$${𝐇}_{\text{final}}=\lambda {𝐇}_{\text{text}}^{\prime }+(1-\lambda ){𝐇}_{\text{image}}^{\prime }$$ (29)

where ${𝐖}_{\text{fc}}$ and ${𝐛}_{\text{fc}}$ are the weight matrix and bias matrix of the linear transformation, respectively; *C* denotes the number of emotion classes; *λ* controls the weighting factor when summing the text and image probability vectors.

### 4.3 Multimodal sentiment classification

For each text-image pair (*T*,*I*) fed into the multimodal sentiment classifier, we obtain a predicted probability distribution **P**. The index of the maximum value in this probability vector is taken as the predicted emotion label $\hat{y}$. The computation proceeds as follows:

$$𝐏=\text{Softmax}({𝐇}_{\text{final}})$$ (30)

$$\hat{y}=\mathrm{argmax}(𝐏)$$ (31)

where **P** is the model’s predicted probability vector over all emotion classes, and $\hat{y}$ is the final predicted emotion label.

During training, the model is optimized with the AdamW optimizer to minimize the sum of cross-entropy loss and Contrastive Loss:

$$Loss=-\sum_{i}y_{i}\log(\hat{p_{i}})+{ℒ}_{\text{contrastive}}$$ (32)

where ${\hat{p}}_{i}$ is the Softmax output and *y*_*i*_ is the ground-truth emotion label; the contrastive loss employs a cosine-embedding loss.

## 5 Evaluation

In the experimental implementation phase, we will introduce the dataset required for the experiment, the baseline algorithms, and the relevant results in sequence.

### 5.1 Datasets

This paper implements experiments with various models on the SDTI dataset to evaluate the performance of the proposed FFBT method. The evaluation metrics include class-specific Precision, Recall, and F1-Score for individual category recognition, along with overall classification metrics including Accuracy, Macro-F1, and Weighted-F1.

The SDTI dataset was constructed by relabeling data from the public MVSA-Single benchmark [42], with all data collection and analysis procedures complying with the source platform’s terms and conditions, and obtaining necessary ethical approvals or user authorizations. The SDTI dataset comprises 5,129 text-image paired samples, with a standardized split of 4,001 samples for training, 616 for validation, and 512 for testing, resulting in a ratio of approximately 8:1:1 between training, validation, and test sets. As shown in Table 2, the emotional label distribution across different categories in the SDTI dataset follows an approximate 6:1:3 ratio.

**Table 2:** The distribution of different sentiment labels in dataset SDTI.

Dataset	Positive	Neutral	Negative	Total
SDTI	3078	545	1506	5129

### 5.2 Baseline

To validate the performance of our proposed FFBT method, we conduct comprehensive comparisons with the following baseline algorithms:

**ResNet-SVM algorithm** [43]: uses CNNs or ResNet series networks to extract text-image features and employs SVM to classify by finding the hyperplane that maximizes the interval between classes in the feature space. For nonlinearly separable data, it maps to a higher-dimensional space through kernel tricks to find the optimal decision boundary.

**CNN-DT algorithm** [44]: uses CNNs to extract features from text and another pre-trained CNN to extract features from images. After simply concatenating the two parts of the features, it uses a Decision Tree to build a tree-like structure for feature classification by recursively selecting the optimal features and splitting points: it starts from the root node, divides the data into different branches according to the feature values, each branch represents a decision rule, and recursively divides the dataset into smaller, purer subsets until reaching the leaf node, with each leaf node corresponding to a classification result.

**BERT-RF algorithm** [45]: proposes using BERT for text feature extraction, while image features are implemented with general CNNs or ResNet. Then, it uses Random Forrest to classify sentiments by constructing multiple decision trees, each of which randomly selects a subset of features and samples during training, and then aggregates the classification results of each tree through a majority voting mechanism.

**DNN-LR algorithm** [46]: leverages two distinct pre-trained CNNs to extract features from text and images, respectively, and subsequently utilizes Logistic Regression for sentiment tendency classification.

**RoBERTa–ClipVisionModel** [47]: concatenates the textual and image features extracted by RoBERTa and CLIP’s Vision Transformer, respectively, and then feeds the fused vector into a multilayer perceptron for sentiment classification.

**MultiSentiNet** [48]: extracts objects and scenes from images as visual-semantic cues and introduces an image-guided LSTM network with attention to extract key textual terms.

**ViLT** [49]: a Transformer-based framework that introduces image-language cross-modal embeddings to jointly relate visual and textual information.

**VAuLT** [50]: an extension of ViLT that improves performance on vision-and-language tasks involving more complex text inputs than image captions, while having minimal impact on training and inference efficiency.

**FFBT (BERT)**: identical to our proposed method except that it uses BERT instead of RoBERTa for text feature extraction.

### 5.3 Implementation details

In the experimental deployment, as mentioned in Sect 5.1, the SDTI dataset will be allocated to the training set, validation set, and test set in a ratio of approximately 8:1:1. The optimizer used by the proposed FFBT method is AdamW. The learning rate is initialized at 5e-6 and reduced with a weight decay ratio of 1e-2. In response to the sample sizes across the training, validation, and test sets, we configured the batch size for the training and validation sets to 16 and for the test set to 8. Our model is implemented based on PyTorch. This paper uses Accuracy, Macro-F1, and Weighted-F1 as performance metrics to comprehensively assess the model, whose definitions are as follows.

$$\text{Accuracy}=\frac{\text{TP}+\text{TN}}{\text{TP}+\text{TN}+\text{FP}+\text{FN}}$$ (33)

$$\text{Precision}=\frac{\text{TP}}{\text{TP}+\text{FP}}$$ (34)

$$\text{Recall}=\frac{\text{TP}}{\text{TP}+\text{FN}}$$ (35)

$$\text{F}1=\frac{2\cdot \text{Precision}\cdot \text{Recall}}{\text{Precision}+\text{Recall}}$$ (36)

Where TP stands for True Positive, TN stands for True Negative, FP stands for False Positive, and FN stands for False Negative.

The macro average refers to the simple average of the metrics for each category, regardless of the support degree (i.e., the number of samples) of each category. The weighted average refers to the weighted average of the metrics based on the support degree of each category.

$$\text{Macro Average}=\frac{1}{N}\sum_{i=1}^{N}M_{i}$$ (37)

$$\text{Weighted Average}=\frac{\sum_{i=1}^{N}(M_{i}\times S_{i})}{\sum_{i=1}^{N}S_{i}}$$ (38)

Where *N* is the number of categories, *M*_*i*_ is the metric value for each category (such as precision, recall, f1-score, etc.), and *S*_*i*_ is the sample size or support degree.

### 5.4 Main results

**Fig 5:**
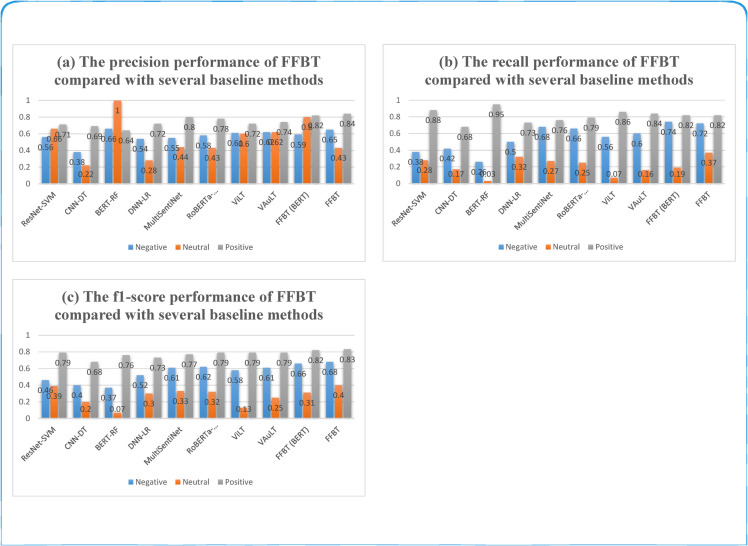
The performance of different methods for individual category recognition.

On the SDTI dataset, the proposed FFBT algorithm is compared with the baseline methods; the results are reported in Fig 5 and Table 3.

**Table 3:** The comparison of different methods on SDTI dataset.

Method	Accuracy	Macro-F1	Weighted-F1
CNN-DT	0.55	0.42	0.55
DNN-LR	0.62	0.51	0.62
BERT-RF	0.64	0.40	0.57
ResNet-SVM	0.68	0.54	0.65
MultiSentiNet	0.688	0.57	0.68
RoBERTa-ClipVisionModel	0.695	0.53	0.65
ViLT	0.69	0.50	0.67
VAuLT	0.70	0.55	0.69
FFBT (BERT)	0.734	0.60	0.72
FFBT	0.741	0.64	0.74

From the results in the above Fig 5 and Table 3, we can observe that: (1) CNN-DT performs the worst because decision trees refine rules via locally optimal splits that can grow overly complex and fit training noise; each split relies on only one feature, making it impossible to capture word-order or negation dependencies such as “not + happy,” leading to low accuracy for subtle emotions like sarcasm. (2) DNN-LR outperforms Decision Tree by about 7%, since Logistic Regression handles high-dimensional, sparse textual features more efficiently. (3) When BERT replaces the DNN for text processing, BERT-RF reaches an even higher score, thanks to BERT’s powerful attention mechanism and Random Forest’s ability to reduce overfitting on textual features. (4) ResNet-SVM uses ResNet to extract fine-grained image features and leverages the kernel trick of SVM to model non-linear relations, achieving comparatively strong benchmark performance. (5) By incorporating visual cues into text, MultiSentiNet improves further. (6) RoBERTa-ClipVisionModel, which concatenates text and image features after Transformer encoding, slightly surpasses MultiSentiNet. (7) ViLT and VAuLT, both built on Transformers with explicit cross-modal learning, achieve the best scores among all baselines. (8) FFBT (BERT) trails FFBT marginally, as RoBERTa is essentially an enhanced version of BERT.

On the SDTI dataset, compared with the strongest baseline, our FFBT model—excluding the RoBERTa-equivalent FFBT (BERT)—improves accuracy, Macro-F1, and Weighted-F1 by 4.1%, 7%, and 5%, respectively. Across all metrics, the results consistently demonstrate the superiority of FFBT. This performance gain stems from three key enhancements: first, RoBERTa’s byte-level Byte-Pair Encoding eliminates out-of-vocabulary issues, greatly expanding the vocabulary and enabling richer textual feature extraction; second, ResNet50’s residual design mitigates gradient explosion, producing robust image representations; and finally, feeding the textual and image features into a multimodal Transformer encoder with cross-modal attention strengthens their semantic interaction, allowing complementary information across modalities and yielding more accurate predictions.

### 5.5 Ablation study

To gain a clearer understanding of the roles of different modules in the proposed model, we organized a series of ablation experiments on the SDTI dataset.

**Fig 6:**
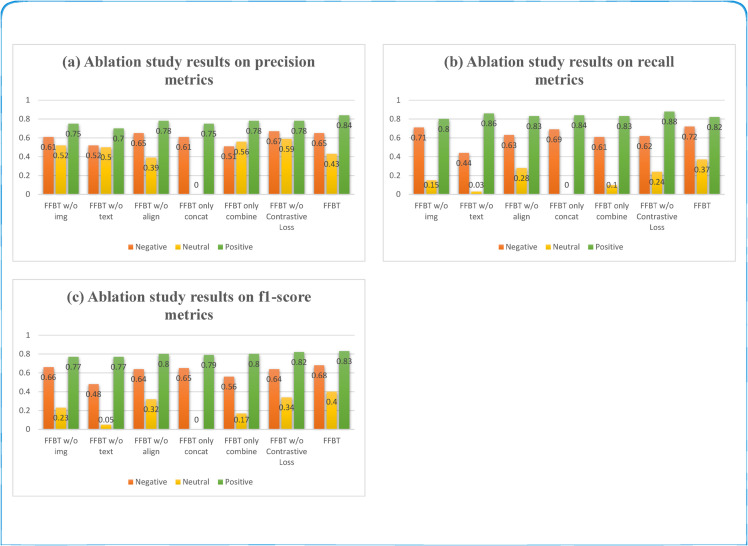
The performance of Ablation Study for individual category recognition.

In this section, we first validate the superiority of multimodal analysis over unimodal analysis by examining text-only (“FFBT w/o img”) and image-only (“FFBT w/o text”) configurations. Subsequently, we investigate the contribution of key components by: (1) removing the cross-modal alignment module (“FFBT w/o align”), (2) replacing the fusion module with simple concatenation (“FFBT only concat”) or element-wise addition (“FFBT only combine”), and (3) eliminating the contrastive loss (“FFBT w/o Contrastive Loss”). The experimental results are presented in Fig 6 and Table 4.

**Table 4:** Ablation Study results on SDTI dataset.

Method	Accuracy	*Δ* v.s. Full	Contribution	Macro-F1	Weighted-F1
FFBT w/o img	0.694	- 4.7%	24.6% ↓	0.55	0.67
FFBT w/o text	0.653	- 8.8%	46.1% ↓	0.43	0.61
FFBT w/o align	0.711	- 3%	15.7% ↓	0.59	0.71
FFBT only concat	0.707	- 3.4%	17.8% ↓	0.48	0.67
FFBT only combine	0.69	- 5.1%	26.7% ↓	0.51	0.67
FFBT w/o Contrastive Loss	0.737	- 0.4%	2.1% ↓	0.60	0.72
FFBT	0.741	-	100%	0.64	0.74
Baseline (CNN-DT)	0.55	- 19.1%	-	0.42	0.55

Where the contribution is defined as:

$$\text{Contribution of Module}X=\frac{\text{Full score}-\text{w/o }X\text{ score}}{\text{Full score}-\text{Baseline score}}\times 100\%$$ (39)

From the ablation studies above, we can observe that:

(1) Due to the higher semantic explicitness of textual data compared to the abstract nature of images—and considering that sentiment classification may rely more heavily on textual content (e.g., sentiment words directly appearing in the text)—the performance metrics of “FFBT w/o img” are consistently higher than those of “FFBT w/o text” by 4.1%. (2) When only textual or image data is used for unimodal sentiment classification (multi-label task), the performance metrics lag behind those of the text-image bimodal approach by 4.7% and 8.8%, respectively. This disparity arises because the combined text-image information enhances the classifier’s recognition capability. (3) The performance degradation of “FFBT w/o align” compared to the full FFBT model underscores the critical role of cross-modal alignment in modeling latent relationships between textual tokens and visual regions for effective multimodal sentiment classification. (4) Both simplified fusion variants exhibited significant limitations: “FFBT only concat” showed –3.4% accuracy degradation, and “FFBT only combine” suffered –5.1% accuracy loss. This empirically confirms that our proposed fusion mechanism enables fine-grained feature interaction, outperforming naive fusion strategies by dynamically learning modality-specific attention weights. (5) After incorporating the contrastive loss from contrastive learning, FFBT achieves better alignment between image and text feature vectors, enabling finer-grained feature interaction. Consequently, its performance metrics slightly outperform those of “FFBT w/o Contrastive Loss”.

### 5.6 Hyper-parameter analysis

In the series of experiments we designed, the number of attention heads *m* in the cross-modal fusion mechanism and the weighting factor *λ* in Eq 29 are two key hyper-parameters that directly affect inference performance. Therefore, this section analyzes the performance of the proposed method under various settings of *m* and *λ*.

**Fig 7:**
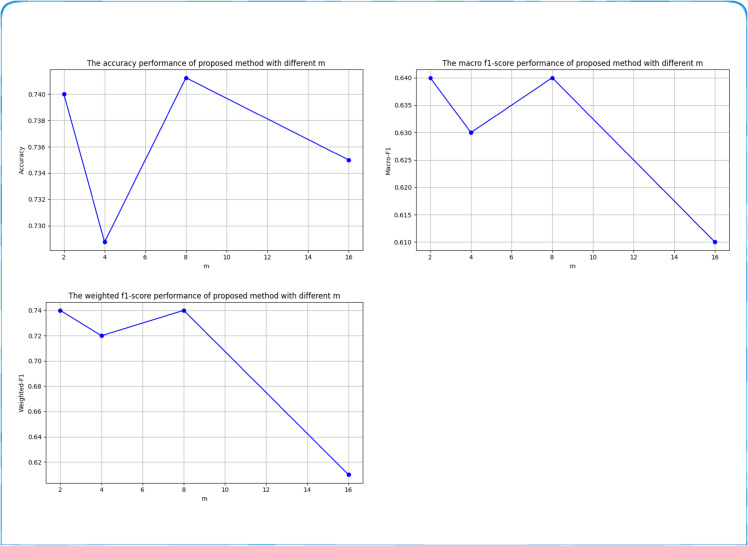
Hyper-parameter Analysis of different number of attention heads m for proposed FFBT.

We first examine how the number of attention heads *m* affects performance. Fig 7 reports results for different *m* values. Setting *m* = 8 yields the best outcome because it aligns the per-head dimension with the fine-grained details required for text–image alignment. Values of 2 or 4 under-fit the data, while 16 heads are too fine-grained, leading to over-fitting and memory bottlenecks. Consequently, we fix *m* = 8 in all subsequent experiments.

**Fig 8:**
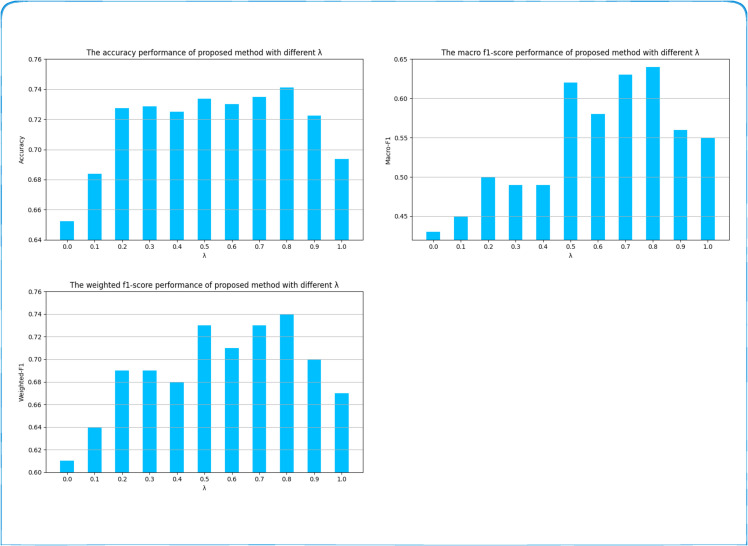
Hyper-parameter Analysis of different *λ* in Eq 29 for proposed FFBT.

We then conduct experiments to investigate the effect of varying *λ* in Eq 29. As shown in Fig 8, setting λ=0.8 yields the best accuracy and F1-score. When only image features are used (λ=0) or only text features are used (λ=1), performance drops. Hence, leveraging both image and textual information is essential for overall emotion classification. Based on these results, we set λ=0.8 in our final method.

## 6 Conclusions

This paper proposes a Feature-Fusion-Based Transformer (FFBT) designed to identify the emotional tendencies expressed by students on social networking platforms, laying the groundwork for future intelligent and personalized psychological support education at all school levels. The method leverages RoBERTa for textual content and ResNet50—a CNN-based architecture with residual connections—for image content extracted from student Weibo posts. A multimodal Transformer encoder then performs cross-modal interaction and fusion, after which fully-connected layers classify the fused features into emotional categories. Experimental results demonstrate that FFBT not only overcomes the limitations of traditional handcrafted features and modality isolation but also exploits the combined power of textual and image information to improve the accuracy of student emotion classification. Considering the exponential growth of social-media data and the increasing complexity of human emotions, future sentiment-analysis systems should move beyond simple positive, negative, and neutral labels to more nuanced states such as guilt, pride, or anger. Consequently, our future work will investigate even more sophisticated feature alignment and fusion strategies to capture these subtle emotional tendencies, continually providing technological support for intelligent and personalized psychological counseling.

## Supporting information

S1 Data The relabeled SDTI dataset derived from the public MVSA-Single benchmark [46].
(ZIP)
